# Sterilizing elastomeric chains without losing mechanical properties. Is it possible?

**DOI:** 10.1590/2176-9451.20.3.096-100.oar

**Published:** 2015

**Authors:** Matheus Melo Pithon, Caio Souza Ferraz, Francine Cristina Silva Rosa, Luciano Pereira Rosa

**Affiliations:** 1 Professor of Orthodontics, Universidade Estadual do Sudoeste da Bahia (UESB), Jequié, Bahia, Brazil; 2 Undergraduate student in Dentistry, Universidade Estadual do Sudoeste da Bahia (UESB), Jequié, Bahia, Brazil; 3 Professor of Microbiology, Universidade Federal da Bahia (UFBA), Vitória da Conquista, Bahia, Brazil; 4 Professor of Radiology, Universidade Federal da Bahia (UFBA), Vitória da Conquista, Bahia, Brazil

**Keywords:** Orthodontics, Disinfection, Elastomers

## Abstract

**OBJECTIVE:**

To investigate the effects of different sterilization/disinfection methods on the mechanical properties of orthodontic elastomeric chains.

**METHODS:**

Segments of elastomeric chains with 5 links each were sent for sterilization by cobalt 60 (Co60) (20 KGy) gamma ray technology. After the procedure, the elastomeric chains were contaminated with clinical samples of *Streptococcus mutans*. Subsequently, the elastomeric chains were submitted to sterilization/disinfection tests carried out by means of different methods, forming six study groups, as follows: Group 1 (control - without contamination), Group 2 (70°GL alcohol), Group 3 (autoclave), Group 4 (ultraviolet), Group 5 (peracetic acid) and Group 6 (glutaraldehyde). After sterilization/disinfection, the effectiveness of these methods, by Colony forming units per mL (CFU/mL), and the mechanical properties of the material were assessed. Student's t-test was used to assess the number of CFUs while ANOVA and Tukey's test were used to assess elastic strength.

**RESULTS:**

Ultraviolet treatment was not completely effective for sterilization. No loss of mechanical properties occurred with the use of the different sterilization methods (p > 0.05).

**CONCLUSION:**

Biological control of elastomeric chains does not affect their mechanical properties.

## Abstract (pt)

**OBJETIVO:**

verificar os efeitos de diferentes métodos de esterilização/desinfecção nas propriedades mecânicas de elásticos ortodônticos em cadeia.

**MÉTODOS:**

segmentos de elástico em cadeia com 5 elos cada foram enviados para esterilização em radiação gama com cobalto 60 (20 KGy). Após esterilização, esses foram contaminados com amostras clínicas de *Streptococcus mutans*. Passado esse período, foram submetidos aos testes de esterilização/desinfecção por diferentes métodos, formando seis grupos de estudo, assim denominados: Grupo 1 (controle - sem ter sido contaminado), Grupo 2 (álcool 70°GL), Grupo 3 (autoclave), Grupo 4 (ultravioleta), Grupo 5 (ácido peracético) e Grupo 6 (glutaraldeído). Após esterilização/desinfecção, avaliou-se a efetividade desses métodos, por meio de contagem de unidades formadoras de colônias por mL (UFC/mL), e as propriedades mecânicas desses materiais. Utilizou-se o teste *t* de Student para avaliar o número de UFC, além do ANOVA e, posteriormente, do teste de Tukey para avaliação da força.

**RESULTADOS:**

verificou-se que o ultravioleta não obteve eficácia total quanto à esterilização. E não ocorreu perda das propriedades mecânicas dos elásticos, com os diferentes métodos de esterilização utilizados (p > 0,05).

**CONCLUSÃO:**

o controle biológico de elásticos em cadeia não interfere nas suas propriedades mecânicas.

## INTRODUCTION

Fighting infections in dental offices has been a daunting challenge to dentists, researchers and immunologists. Most of times, germs have been able to dodge contemporary safety measures, thereby exposing professionals and patients to risk. On the other hand, lack of care by some professionals with regard to biosafety has favored the intensification of infection.^1,2^

Of the dental specialties, Orthodontics is outstanding among those with a higher number of predisposing factors for cross-infection.^3,4^ Orthodontics is characterized by a high turnover of patients and multiplicity of vehicles for disease transmission (equipment, instruments, operators' hands, etc.), thus exposing clinicians, assistants and patients to serious risks of infection.^5,6^

In Orthodontics, elastomeric chains are among the different types of material that highly favor the occurrence of cross-infection, given that this type of material is commercially presented in reels ranging from 1 to 4.5 meters, which hinders its individual use.^7,8^

Despite wide acceptance and use of elastomeric chains, doubt is cast on their mechanical and biological properties after they have been submitted to sterilization procedures.^9^ Considering that elastics and elastomeric chains are amorphous polymers made of polyurethane material, presenting characteristics of both rubber and plastic, their characteristics may be altered in contact with physical and or chemical agents.^10^

Thus, the present study aimed at assessing which method would be most indicated to sterilize elastomeric chains without causing them to lose their mechanical properties.

## MATERIAL AND METHODS

Elastomeric chains (Morelli, Sorocaba, Brazil) of the short spacing type were carefully removed from the reel without being elongated/stretched, and cut into segments with 5 links each. Subsequently, they were wrapped in surgical grade paper (n = 15) and sent to sterilization by gamma radiation with cobalt 60 (20 KGy) (Empresa Brasileira de Radiação - EMBRARAD, Cotia-SP, Brazil) without alterations in their physical properties.

## Assessing effectiveness of different methods

After specimens were sterilized, they were contaminated in test tubes containing 10 mL of TODD liquid culture medium with 100 microliters of standardized suspension for assessment by spectrophotometry (optical density = 0.620; wavelength = 398) of 1 X 10^6^ cells/mL of ten different randomly selected clinical samples of *Streptococcus mutans*. Specimens were then incubated at 37 ^o^C for 48 h**.**

After the incubation period and *Streptococcus mutans *monospecies biofilm formation adherent to the specimens, the latter were introduced into polypropylene tubes, containing 2 mL of sterile saline solution (0.85% NaCl), for 10 seconds, so as to remove excess biofilm. Specimens were then introduced into appropriate and sterile receptacles so as to be subjected to sterilization tests, as follows:

» Group 1: Elastomeric chains which were not submitted to any sterilization method (control group).
» Group 2: Elastomeric chains immersed in polypropylene tubes containing 2 mL of 70° GL alcohol for 1 minute.
» Group 3: Elastomeric chains autoclaved for a cycle of 15 minutes.
» Group 4: Elastomeric chains sterilized in ultraviolet light (SPLabor, Presidente Prudente, São Paulo, Brazil) for 30 minutes, divided by 15 minutes on each side of the elastic.
» Group 5: Elastomeric chains immersed in polypropylene tubes containing 2 mL of peracetic acid for 30 minutes.
» Group 6: Elastomeric chains immersed in polypropylene tubes containing 2 mL of 2% glutaraldehyde solution for 30 minutes.

After sterilization/disinfection procedures were carried out by the different methods, the specimens were removed in a sterile environment inside a laminar flow chamber and introduced into polypropylene tubes containing 2 mL of sterile saline solution (0.85% NaCl), agitated in a vortex appliance for 1 minute. From the suspension obtained, decimal dilutions of 10^-^ 1, 10^-^ 2 were made. Aliquots of 100 microliters of initial suspension and the other dilutions were seeded on Petri dishes containing Todd Hewitt broth at 37 ^o^C for 48 h.

Subsequently, each dish was examined by a single previously calibrated investigator to determine the number of colony forming units per mL (CFU/mL) with the aid of a colony counter (CP602, Phoenix, Araraquara, São Paulo, Brazil).

## Assessing mechanical properties

After being submitted to different biological control methods, the strength generated by the elastomeric chains was measured (n = 15)according to the previously established sequence of groups.

Elastomeric chains were taken to a digital dynamometer (Instrutherm DD-300, São Paulo, Brazil) mounted on a platform specifically set up for this investigation. Elastomeric chains were distended for 23.5 cm.

## Statistical analysis

After assessing the number of colonies formed and the maximum values obtained by the elastomeric chains, statistical analyses were carried out. To this end, SPSS 13.0 software (SPSS Inc., Chicago, Illinois, USA) was used. Descriptive statistical analysis including mean and standard deviation was carried out for all groups. The values referring to the number of colonies formed were submitted to Student's *t*-test with a significance level set at 5%. The values referring to the amount of strength released were submitted to analysis of variance (ANOVA) so as to determine whether there were statistical differences among groups. Tukey's test was later performed.

## RESULTS

Results referring to the mean number of colony forming units (CFU/mL) reveal that the control group obtained the highest mean of around 220,000 CFU/mL, whereas the group in which ultraviolet light (UV) was used as the method for microorganism control obtained an approximate mean of 80,000 CFU/mL.

**Figure 1.**
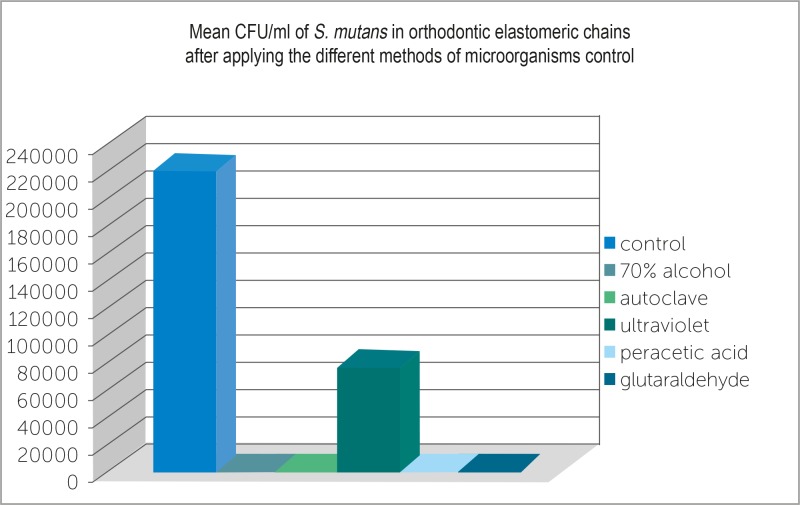
Mean CFU/mL of S. Mutans on orthodontic elastomeric chains after applying the different methods of microorganisms control.

When UV was compared to the other biological control methods, it proved to be the least effective in reducing microorganisms (p = 0.010) (Table 1). There were statistical differences between the control group and the other groups (p < 0.05) (Fig 1 and Table 2).

**Table 1.** Methods with description of respective groups.

	Sterilization method	Time	Pressure	Volume	Temperature
Group 1	-	-	-	-	-
Group 2	70° GL alcohol	1 min	-	1 mL	Room
Group 3	Autoclave	15 min	1 atm	-	121 °C
Group 4	Ultraviolet	15 min p/surface	-	-	Room
Group 5	Peracetic acid	30 min	-	2 mL	Room
Group 6	Glutaraldehyde	30 min	-	2 mL	Room

**Table 2.** Mean, standard deviation and statistical analysis of the number of colony forming units for the different groups evaluated.

Group	Biological control methods	Mean (SD)	Statistics
1	Control	220133.2 (53911.093)	-2/p = 0.000*
			-3/p = 0.000*
			-4/p = 0.000
			-5/p = 0.000
			-6/p = 0.000
2	70° GL alcohol	0.00 (0)	-3/p = 1.000
			-4/p = 0.010*
			-5/p = 1.000
			-6/p = 1.000
3	Autoclave	0.00 (0)	-4/p = 0.010*
			-5/p = 1.000
			-6/p = 1.000
4	Ultraviolet	75956 (83643)	-5/p = 0.010*
			-6/p = 0.010*
5	Peracetic acid	0.00 (0)	-6/p = 1.010
6	Glutaraldehyde	0.00 (0)	

SD = standard deviation;

* = statistical differences (p < 0.05).

With regard to the percentage of decontamination of elastomeric chains, the UV group obtained the lowest percentage of around 65%, whereas the other methods obtained 100%.

In terms of mechanical properties, no differences were found among the different sterilization methods (p > 0.005) (Table 3).

**Table 3.** Statistics of different biological control methods in terms of evaluation of the mechanical properties of elastomeric chains.

Groups	Methods of sterilization	Mean (SD)	p value
1	Control	6.36 (0.79)	-2/p = 0.571
			-3/p = 1.000
			-4/p = 0.478
			-5/p = 0.810
			-6/p = 0.997
2	70° GL alcohol	5.74 (0.84)	-3/p = 0.370
			-4/p = 0.012
			-5/p = 0.999
			-6/p = 0.292
3	Autoclave	6.48 (0.85)	-4/p = 0.686
			-5/p = 0.618
			-6/p = 1.000
4	Ultraviolet	7.02 (1.03)	-5/p = 0.036
			-6/p = 0.771
5	Peracetic acid	5.89 (1.21)	-6/p = 0.524
6	Glutaraldehyde	6.53 (1.11)	

SD = standard deviation.

* = statistical differences (p < 0.05).

## DISCUSSION

When manipulating orthodontic elastomeric chains at the time of inserting them into patient's oral cavity, the orthodontist indirectly contaminates the reel that contains the material which may trigger a cross-infection.

Cross-infection is defined as the transmission of infectious agents among patients and health personnel within a clinical environment. Transmission occurs from person to person or by contact with contaminated objects. Transmission may occur through blood, saliva droplets, or instruments contaminated with blood, saliva and tissue debris. Transmission pathway is either by contact, inhalation or inoculation.^1^

According to Silva et al,^11^ there is a high incidence of cross-infection in the dental office. Thus, the use of decontaminating agents is relevant in clinical practice. A number of methods is used in the dental office with a view to dodging cross-infection, namely: autoclave, alcohol, glutaraldehyde, peracetic acid and ultraviolet radiation.

According to Berger,^12^ ultraviolet radiation (UV) is used in Dentistry as a disinfectant agent for toothbrush surfaces; however, its effectiveness is greatly related to the time of exposure. In the present study, ultraviolet radiation obtained the lowest percentage (65%) in the reduction of colony forming units (CFU/mL) in comparison to the other groups in which disinfectant agents were used. The latter reduced CFUs/mL in 100%. When the mechanical properties of elastomeric chains were compared, UV obtained the best mean, around 7.02; however, without significant differences among groups.

In the present study, glutaraldehyde proved an efficient disinfectant agent, in addition to not affecting the mechanical properties of elastomeric chains, given that there was no significant difference between this group (6.53) and the control group (6.36). These data corroborate the findings by Suprono et al^13^who reported that glutaraldehyde does not cause deterioration of the elastomeric chain surface.

Peracetic acid has been used in food and water industries, sewage treatment companies and for decontamination and sterilization of heat-sensitive medical-hospital devices and equipment.^14^^-^ 17 Peracetic-acid proved an efficient decontaminating agent and completely reduced the number of colony forming units (CFU/mL). Furthermore, peracetic acid does not leave residues and does not produce harmful products, as its mechanism of action involves the release of free oxygen and hydroxyl radicals in decomposition in water, oxygen and acetic acid.^14^^-^ 17 This was proved in the present study, since all elastomeric chains evaluated kept their mechanical properties, in addition to being completely sterilized.

The method most used for sterilization of medical and dental instruments worldwide is damp steam sterilization (autoclave).^18^ It proved efficient in reducing the number of colony forming units (CFU/mL), thereby completely reducing the existent bacteria. Moreover, it yielded surprising results in terms of the mechanical properties of elastomeric chains, since even in contact with heat, the mechanical properties remained the same, without statistical differences in comparison to the control group.

Based on the results obtained in this study, the simplest method of promoting sterilization/disinfection of orthodontic elastomeric chains was alcohol. After 1 minute, it was possible to eliminate the microorganisms adhered to the elastics without losing their mechanical characteristics. Nevertheless, the fact that only *S. Mutans* was used in the experiment must be considered. In spite of being the most prevalent and most important infectious agent in the oral cavity, this bacterium is not the most resistant; therefore, further studies are warranted to investigate other microorganisms.

Importantly, orthodontic clinic success not only involves mastery of corrective techniques to achieve the ideal dental occlusion, but also requires the application of biosafety rules and concerns about the local and systemic consequences of dental material used for this purpose.

## CONCLUSION

Based on the results of this study it is reasonable to conclude that except for the ultraviolet method, all other methods promoted sterilization of elastomeric chains; no sterilization methods led to loss of elastomeric chains mechanical properties.
